# Low rate of capsular contracture in a series of 214 consecutive primary and revision breast augmentations using microtextured implants

**DOI:** 10.1016/j.jpra.2017.10.007

**Published:** 2017-12-27

**Authors:** Brent Tanner

**Affiliations:** Spire Hospital, Fordcombe Road, Tunbridge Wells, Kent TN3 0RD, UK

**Keywords:** Breast augmentation, Breast implant, Capsular contracture, Texture

## Abstract

Capsular contracture is the most common major complication after implant-based breast augmentation. The aetiology of capsular contracture is multifactorial. The author reports a retrospective personal series of patients managed over a seven-year period with a nearly unchanged surgical strategy implementing most of known measures for capsular contracture prevention. A microtextured silicone gel-filled implant from a single manufacturer was used in all cases.

There were 214 consecutive patients (126 primary augmentations and 88 revision augmentations) operated on over the study period. Mean age of the population was 40.0 years, and mean BMI was 22.0 kg/m^2^. Of the patients in the revision cohort, 44.3% were reoperated on because of previous capsular contracture.

Average follow-up was 20.2 months. There was a 0% capsular contracture rate in the primary augmentation cohort and a 3.4% capsular contracture rate in the revision cohort. At last follow-up, 91.2% of breasts received a Baker I grading.

Although the follow-up was relatively short, this rate of capsular contracture would still be considered very low. Determining the reason for such a low rate of capsular contracture on Multivariate Analyses would be difficult due to the potential myriad of confounding variables. However, given the constancy of the technique and implant type employed by a single surgeon, the author is of the opinion that the microtexturing topography on the implant surfaceused in this series contributed to the low rate of capsular contracture formation. However, this would need to be tested in arandomized controlled trial comparing microtextured devices with implants that have macrotextured surfaces.

## Introduction

Implant-based breast augmentation is the most frequently performed cosmetic surgery worldwide.^1^ The most common significant complication after breast augmentation is capsular contracture (CC). In US Food and Drug Administration (FDA) premarket approval studies, which are performed with undisputable scientific rigour and large sample sizes, 10-year postoperative CC rates were found to be as high as 18.9% for primary cases and 28.9% for revision cases.^2^, ^3^, ^4^, ^5^

For many years, experimental and clinical trials have attempted to clarify the aetiology of and preventative techniques for CC. Debate continues on various proposed causes of CC, including haemorrhage with blood accumulation in the pocket, bacterial contamination, foreign body reaction to necrotic tissue, gel bleed and/or inadequate pocket dissection.^6^, ^7^

It is generally agreed that the aetiology of CC is multifactorial. However, because of their design, most clinical studies fail to consider the significance of each of the possible causal factors. Most clinical trials with large populations are multicentred and include cohorts with different surgical strategies represented. Few reports have contained populations who underwent the same surgical procedure with the same implant type.

The author postulates that the importance of implant characteristics (particularly, implant surface topography) is underestimated for the prevention of CC.

The author reports a retrospective personal series of 424 augmented breasts in 214 consecutive patients operated on over a 7-year period with a nearly unchanged surgical strategy and a microtextured silicone gel-filled implant from a single manufacturer.

## Materials and methods

Retrospective analysis was performed for all patients (n = 214) operated on by the author from February 2009 to August 2016. Both primary augmentation (n = 126) and revision augmentation (n = 88) were performed. All the implants used were the same: textured, silicone gel-filled, round implants (LS 90, LSC 92, or LSC 93; Groupe SEBBIN SAS; Boissy l'Aillerie, France).

Surgeries were performed under general anaesthesia. Perioperative antibiotics (gentamicin 160 mg IV and cefuroxime 1.5g) were administered. Patients were placed in the supine position on the operating table with arms extended. The skin in the surgical area was cleansed with iodine or chlorhexidine in alcohol, and plastic film nipple guards were applied. Local anaesthesia (0.25% marcaine with 1:200,000 epinephrine, 20 ml per breast) was used. Inframammary incisions were followed by dissection with a harmonic scalpel in primary cases, and dissection and capsulectomy with scissors and monopolar diathermy in secondary cases. Implants were washed in sterile water only until November 2015; they were subsequently washed with 80 mg of gentamicin and 1.5g of cefuroxime in 500 ml of sterile water. Subfascial or dual plane implant placement was performed without the use of an introduction device. Wound closure was achieved with 3.0 vicryl for the superficial fascia and 4.0 polydioxanone for subcuticular sutures, followed by tissue glue and suture strips superficially. Surgical drains were left in place for a period of 24 hours. No postoperative antibiotics were given.

Elastic abdominal binders were placed on chest for 24 hours to minimize oedema and bruising. Implant stabilizers were used to position the implants for two weeks.

Patients were instructed to wear sports bras with bra extenders to alleviate inframammary tightness. Follow-up consisted of weekly visits for 3 weeks, and then yearly. Patients received consultation once a year free of charge. Only actual visits were considered in calculating the length of the follow-up. Telephone or written communications were not considered because of their unreliability.

Data was retrieved on patient history, surgical details, implant type, and complications. When a patient had more than one surgery performed during the study period by the author (for example, primary surgery and later revision), only the first procedure was included in order to keep the sample composed of independent individuals.

Complications were individually reported as absolute rates, according to patients rather than implants, as requested by the U.S. Food and Drug Administration for their premarket approval studies.

Statistical analysis was performed using Medcalc software (MedCalc Software bvba, Acacialaan, Belgium).

## Results

From February 2009 to August 2016, 214 consecutive patients had breast augmentation performed by the author. Of these, 126 (58.9%) were primary augmentation and 88 (41.1%) were revision surgery. Patient demographics and surgical details are given in Table 1.

**Table 1:** Patients demographics and surgical details.

Patient Demographics	Primary	Revision	Overall
Number of patients	126	88	214
Mean Age *(SD)*	35.4 *(10.7)* years	46.6 *(12.1)* years	40.0 *(12.6)* years
Mean BMI *(SD)*	21.9 *(2.7)* kg/m^2^	22.3 *(3.8)* kg/m^2^	22.0 *(3.2)* kg/m^2^
Smokers	22.2%	18.2%	20.6%
Had children at time of surgery	65.9%	73.8%	69.1%
Preoperative bra cup size (A/B/C/D/E/F/G)	41%/28%/19%/6%/5%/1%/0%	28%/26%/23%/14%/3%/4%/2%	36%/27%/20%/10%/4%/2%/1%

Surgery	Primary	Revision	Overall
Implant volume *(SD)*	337.8 *(65.8)* ml	397.1 *(137.1)* ml	361.8 *(104.8)* ml
Subfascial / Dual plane	88.9%/11.1%	83.3%/16.7%	86.4% /13.6%
Implant washed with sterile water / antibiotics	86.4% / 13.6%	92.9% / 7.1%	89.0% / 11.0%
Use of drain	100%	100%	100%
Surgery duration *(SD)*	71.8 *(42.6)* min	80.5 *(55.5)* min	75.4 *(48.4)* min

Follow-Up	Primary	Revision	Overall
Duration of follow-up *(SD)*	17.1 *(21.4)* months	24.3 *(24.7)* months	20.2 *(23.1)* months

Mean age at the time of surgery was 40.0 y, and mean BMI was 22.0 kg/m^2^. Active smokers made up 20.6% of the patients overall. The reason for primary implantation was cosmetic breast enlargement in 96.3% of the patients, breast asymmetry in 2.8% and tuberous breast anatomy in 0.9%. Implants were washed prior to implantation with only sterile water in 89.0% of cases, and with gentamicin and cefuroxime in sterile water in 11.0% of cases. Implant placement was subfascial in 86.4% of patients and dual plane in the rest. Concomitant surgeries were performed in 6.0% of patients and included abdominoplasty, mastopexy, fat grafting, rhinoplasty, eyelid surgery, facelift surgery, liposuction and thelioplasty. There were no perioperative complications in the series. The mean surgery duration was 75.4 min.

**Figure 1:**
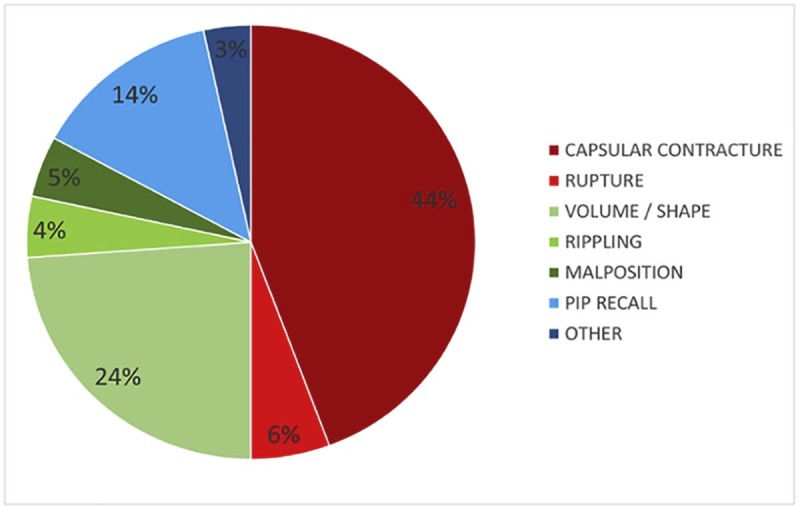
Indications for the 88 revision surgeries.

Figure 1 depicts the indications for the 88 revision surgeries. A total of 39 patients were reoperated on for CC. It was at least a second occurrence of CC for 6 of them.

Out of the 88 patients who had revision surgeries, the removed implants were Silimed implants in 25 patients, PIP implants in 14 patients, Inamed/McGhan implants in 7 patients, Perthese implants in 7 patients, Nagor implants in 3 patients, Mentor implants in 2 patients, and Dow Corning implants in 2 patients. The implant brand could not be retrieved in 28 cases.

The average follow-up was 20.2 months. Table 2 depicts the complications reported as absolute rates.

**Table 2:** Absolute rates of complications.

Complications (per patient)	Primary (N = 126)	Revision (N = 88)	Overall (N = 214)
Rippling	8 (6.3%)	10 (11.4%)	18 (8.4%)
Neuropathic pain	5 (3.9%)	2 (2.3%)	7 (3.3%)
Hematoma	0 (0%)	4 (4.5%)	4 (1.9%)
Implant displacement	1 (0.8%)	1 (1.1%)	2 (0.9%)
Seroma	1 (0.8%)	1 (1.1%)	2 (0.9%)
Deep venous thrombosis	1 (0.8%)	1 (1.1%)	2 (0.9%)
Implant malposition	0 (0%)	1 (1.1%)	1 (0.5%)
Capsular contracture baker iii	0 (0%)	2 (2.3%)	2 (0.9%)
Rupture	1 (0.8%)	0 (0%)	1 (0.5%)
Asymmetry	1 (0.8%)	0 (0%)	1 (0.5%)

Table 3 depicts the capsular contracture Baker grade for the patient population at last follow-up. There were no statistically significant differences in the complication rates (p = 0.6) or the CC grades (p = 0.2) between patients who had implants washed with sterile water and those who had implants washed with antibiotics.

**Table 3:** Capsular contracture grade at the last follow-up visit.

Baker grade	Primary (N = 126)	Revision (N = 88)	Overall (N = 214)
Grade I	96.8%	83.0%	91.2%
Grade II	3.2%	14.7%	7.9%
Grade III	0%	2.3%	0.9%
Grade IV	0%	0%	0%

There was no Baker III or Baker IV CCs in the primary augmentation cohort. In the revision cohort, two patients had unilateral Baker III CCs. The two patients had been reoperated on because of previous CC.

There was one implant rupture. Explant analysis showed evidence of rupture provoked by a surgical instrument.

## Discussion

CC remains the most frequent major complication after breast augmentation. Rates of Baker III and IV CC can reach nearly 20% after primary augmentation and nearly 30% after revision augmentation.^2^, ^3^, ^4^, ^5^

Several authors have been evaluating potential methods for prevention of CC after breast augmentation. Biofilm is a frequently cited cause of CC.^8^, ^9^ Some authors have recommended the use of nipple shields in order to limit the risk of implant contamination.^10^ Nipple shields were used for all patients in the present series. The role of antibiotics in the prevention of CC is debated. Few data are available on the effect of perioperative antibiotic administration on CC formation. However, in the present series, perioperative antibiotics were administered to all patients because they have been demonstrated to reduce the risk of infection.^7^ A few authors have suggested that topical antibiotic irrigation may reduce the risk of CC.^11^ In the present study, 89.0% of implants were washed with sterile water, because of water's bacteriolytic properties, whereas 11.0% were washed with antibiotics and sterile water. There were no significant differences in CC Baker grades between the two techniques. Postoperative oral antibiotics were not prescribed in the present series because there is a lack of evidence in the literature that they further reduce the risk of CC or infection.^12^

The literature on the use of postoperative drainage is sparse, however, an increased CC rate has been demonstrated in cases involving hematoma formation.^13^ Some authors have reported on the efficacy of drainage use to lower the risk of CC.^14^, ^15^ Fanous *et al.* reported a series of 319 primary bilateral augmentations managed with the routine use of drains that had no cases of CC.^14^ Drains were thought by the author to be most important in the prevention of CC, but there was no control group in the study. Surgical drains remained in place for 24 hours for all patients in the present study. High vacuum Redivac drains (Atrium Medical, Hudson, NH, USA) were used. The purpose of drain use was to drain any fluid around the implant and, more importantly, to create a vacuum in the cavity for close adherence of the implant and the surrounding tissues.

Inframammary incision and submuscular placement have been consistently shown to reduce the risk of CC.^16^, ^17^ Although all patients in the present study had inframammary incisions, only 13.6% had partial submuscular (dual plane) placements.

The author believes implant characteristics have a major, but understudied, role in the occurrence of CC. Several findings in the literature support this hypothesis. First of all, gel bleed across the implant has been shown to be a major contributor in the occurrence of CC.^18^ Even with the addition of a low bleed barrier to the shell (done by all manufacturers), a tiny amount of gel may bleed through the envelope; this quantity may vary depending on the implant type and generation.

Another important factor is implant surface. The use of implants with textured surfaces has been clearly shown to reduce the rate of CC.^16^, ^17^, ^19^ However, the author believes that considering “textured implants” as a single entity separate from “smooth implants” is methodologically incorrect. Topographically, all textured breast implants are different. Because they show differences in the extent to which they enable fibroblast adhesion,^20^, ^21^ it seems reasonable that they would be associated with different rates of CC.

The Siltex^®^ (Mentor, Santa Barbara, California, USA) and Biocell^®^ (Allergan, Irvine, California, USA) implants are overrepresented in the literature on textured breast implants because of their long-time presence in the American market.^22^ Despite similar study designs and patient populations in their FDA premarket approval studies, the two manufacturers have reported significant differences in their rates of CC,^2^, ^4^ supporting that implant-specific characteristics may play a major role in CC rates.

In the present study, the author used a standardized surgical strategy in 214 consecutive patients. To the best of the author's knowledge, this is the first report on the clinical use of SEBBIN round silicone gel-filled breast implants. The surface of this implant was described by Danino *et al.* as having pores of 300 to 500 microns in diameter and 10 to 30 microns in depth,^23^ giving it a less pronounced texture roughness compared with Siltex^®^ and Biocell^®^ implant textures. The Biocell^®^ texture actually shows pores of 600 to 800 microns in diameter and 150 to 200 microns in depth,^23^ while the Siltex^®^ texture is in a nodular form with nodule sizes between 50 and 300 microns and variations of altitude between peak and valleys of 250 to 300 microns.^20^

**Figure 2:**
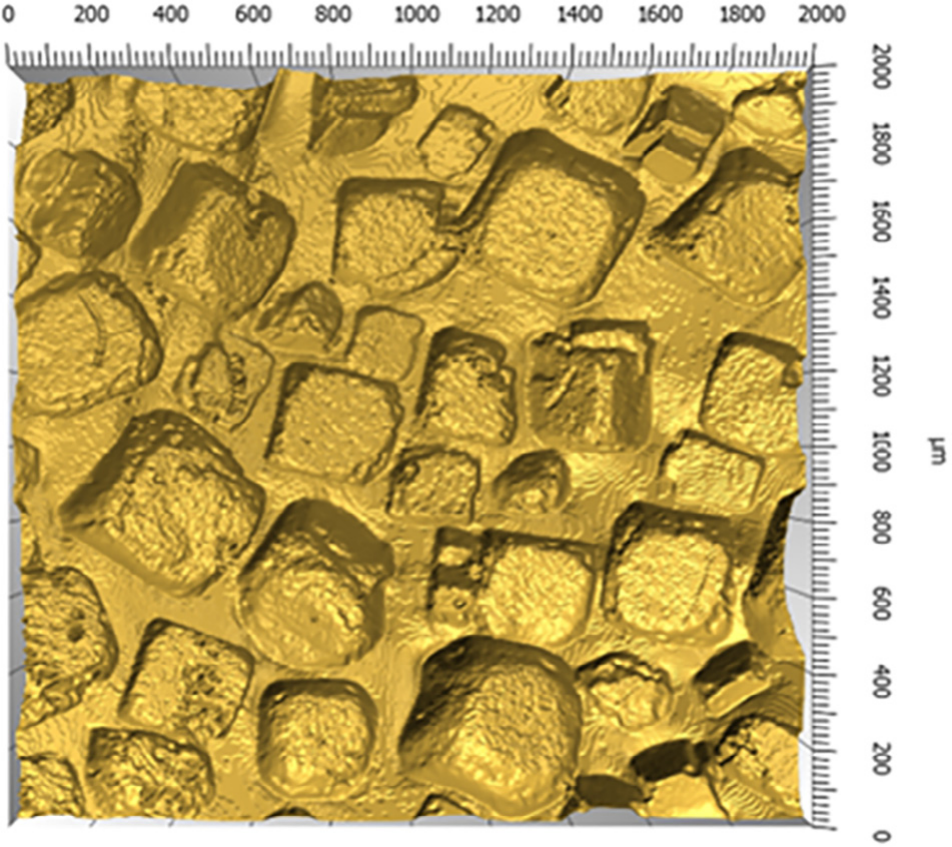
SEBBIN LSC92 implant 4 mm^2^ shell sample visualized using microtomography. Courtesy of LAMIH laboratory (Valenciennes, France).

Figure 2 shows the 3D topography of a 4 mm^2^ sample of a SEBBIN LSC92 implant.

**Figure 3:**
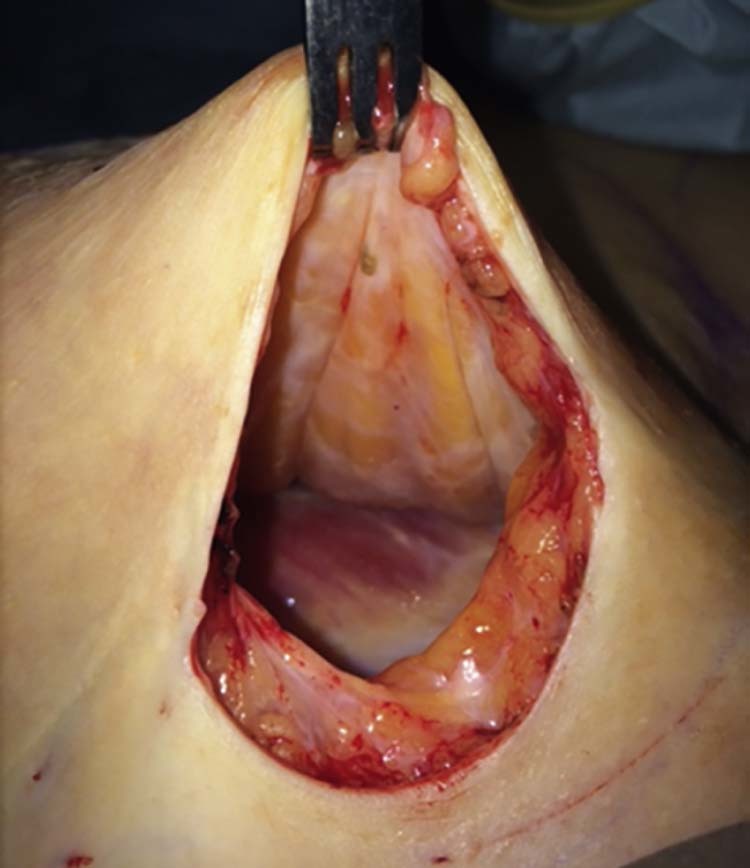
Aspect of the surgical pocket five years post-implantation. Minimal capsular formation.

Using these implants and the most commonly recommended prophylaxis regimen, the author did not identify any cases of CC out of 126 primary augmentations, despite the vast majority of them having a subfascial plane implant placement. Even more surprisingly, the author only recorded two Baker III CCs (2.3%) out of 88 consecutive patients with revision surgeries, 39 of whom were reoperated on because of previous CC. Finally, 91% of all breasts had normal softness (Baker I grading) when they were last examined. These rates of CC are much lower than the rates reported by larger multicentre studies.^2^, ^3^, ^4^, ^5^ Figure 3 shows a lack of capsular formation in a SEBBIN LSC92 implant removed after five years for cosmetic reasons. The patient did not desire implant replacement.

No patient presented with a double capsule in the group of 88 patients requiring revision surgery. This might be because only few patients had previously undergone breast augmentation with macrotextured implants. Similarly, no double capsule was diagnosed during the follow-up of these 214 patients operated on using SEBBIN microtextured implants.

The author believes that a randomized study looking specifically at the rates of CC with the use of different types of textured implants would be helpful to better understand the role of implant characteristics (particularly, implant texture) in the prevention of CC.

Obviously, this study has some limitations. There was no control group, and the average follow-up duration (20.2 months) was quite short. However, it has been demonstrated that 79% of CCs occur during the first two years after surgery.^24^ Moreover, it is unlikely that patients with CC would not have returned to the author for follow-up, especially with free annual consultations. Observer bias cannot be neglected but it is minimized by the author's long experience.

In conclusion, the author reports a personal series of 214 consecutive patients who had primary or revision implant-based breast augmentation with a standardized surgical protocol and microtextured silicone gel-filled implants from a single manufacturer. In the revision cohort, 44.3% of patients were reoperated on because of previous CC. After a follow-up of 20.2 months, the author found a 0% rate of CC in the primary cohort and a 2,3% rate in the revision cohort. Although the follow-up was relatively short, this rate of capsular contracture would still be considered very low. Despite limitations related to the study design, the author is of the opinion that the microtexturing topography on the implant surface used in this series contributed to the low rate of capsular contracture formation. However, this would need to be tested in a randomized controlled trial comparing microtextured devices with implants that have macrotextured surfaces.

## Conflict of interest statement

The author did not receive funding for this study nor any consultancy fee from SEBBIN. He was invited to SEBBIN's manufacturing facility in 2016 with other surgeons. The article processing charges were paid for by SEBBIN.

## References

[1] International Society of Aesthetic Plastic Surgery (2016). ISAPS international survey on aesthetic/cosmetic procedures performed in 2015. http://www.isaps.org.

[2] Caplin D.A. (2014). Indications for the use of MemoryShape breast implants in aesthetic and reconstructive breast surgery: long-term clinical outcomes of shaped versus round silicone breast implants. Plast Reconstr Surg.

[3] Maxwell G.P., Van Natta B.W., Bengtson B.P., Murphy D.K. (2015). Ten-year results from the Natrelle 410 anatomical form-stable silicone breast implant core study. Aesthet Surgery J.

[4] Spear S.L., Murphy D.K., Allergan Silicone Breast Implant USCCSG (2014). Natrelle round silicone breast implants: core Study results at 10 years. Plast Reconstr Surg.

[5] Stevens W.G., Calobrace M.B., Harrington J., Alizadeh K., Zeidler K.R., d'Incelli R.C. (2016). Nine-year core study data for Sientra's FDA-approved round and shaped implants with high-strength cohesive silicone gel. Aesthet Surg J.

[6] Steiert A.E., Boyce M., Sorg H. (2013). Capsular contracture by silicone breast implants: possible causes, biocompatibility, and prophylactic strategies. Med Devices (Auckl).

[7] Chong S.J., Deva A.K. (2015). Understanding the etiology and prevention of capsular contracture: translating science into practice. Clin Plast Surg.

[8] Ajdic D., Zoghbi Y., Gerth D., Panthaki Z.J., Thaller S. (2016). The relationship of bacterial biofilms and capsular contracture in breast implants. Aesthet Surg J.

[9] Rieger U.M., Mesina J., Kalbermatten D.F. (2013). Bacterial biofilms and capsular contracture in patients with breast implants. Br J Surg.

[10] Wixtrom R.N., Stutman R.L., Burke R.M., Mahoney A.K., Codner M.A. (2012). Risk of breast implant bacterial contamination from endogenous breast flora, prevention with nipple shields, and implications for biofilm formation. Aesthet Surg J.

[11] Huang N., Liu M., Yu P., Wu J. (2015). Antibiotic prophylaxis in prosthesis-based mammoplasty: a systematic review. Int J of Surg.

[12] Mirzabeigi M.N., Mericli A.F., Ortlip T. (2012). Evaluating the role of postoperative prophylactic antibiotics in primary and secondary breast augmentation: a retrospective review. Aesthet Surg J.

[13] Handel N., Cordray T., Gutierrez J., Jensen J.A. (2006). A long-term study of outcomes, complications, and patient satisfaction with breast implants. Plast Reconstr Surg.

[14] Fanous N., Salem I., Tawile C., Bassas A. (2004). Absence of capsular contracture in 319 consecutive augmentation mammaplasties: dependent drains as a possible factor. Can J Plast Surg.

[15] Hipps C.J., Raju R., Straith R.E. (1978). Influence of some operative and postoperative factors on capsular contracture around breast prostheses. Plast Reconstr Surg.

[16] Namnoum J.D., Largent J., Kaplan H.M., Oefelein M.G., Brown M.H. (2013). Primary breast augmentation clinical trial outcomes stratified by surgical incision, anatomical placement and implant device type. J Plast Reconstr Aesthet Surg.

[17] Stevens W.G., Nahabedian M.Y., Calobrace M.B. (2013). Risk factor analysis for capsular contracture: a 5-year Sientra study analysis using round, smooth, and textured implants for breast augmentation. Plast Reconstr Surg.

[18] Moyer H.R., Ghazi B.H., Losken A. (2012). The effect of silicone gel bleed on capsular contracture: a generational study. Plast Reconstr Surg.

[19] Pollock H. (1993). Breast capsular contracture: a retrospective study of textured versus smooth silicone implants. Plast Reconstr Surg.

[20] Atlan M., Bigerelle M., Larreta-garde V., Hindie M., Heden P. (2016). Characterization of breast implant surfaces, shapes, and biomechanics: a comparison of high cohesive anatomically shaped textured silicone, breast implants from three different manufacturers. Aesthet Plast Surg.

[21] Valencia-Lazcano A.A., Alonso-Rasgado T., Bayat A. (2013). Characterisation of breast implant surfaces and correlation with fibroblast adhesion. J Mech Beh Biomed Mater.

[22] Wong C.H., Samuel M., Tan B.K., Song C. (2006). Capsular contracture in subglandular breast augmentation with textured versus smooth breast implants: a systematic review. Plast Reconstr Surg.

[23] Danino A., Rocher F., Blanchet-Bardon C., Revol M., Servant J.M. (2001). [A scanning electron microscopy study of the surface of porous-textured breast implants and their capsules. Description of the “velcro” effect of porous-textured breast prostheses]. Ann Chir Plast Esthet.

[24] Kjoller K., Holmich L.R., Jacobsen P.H. (2001). Capsular contracture after cosmetic breast implant surgery in Denmark. Ann Plast Surg.

